# An In-Depth Assessment of the Electronic and Magnetic Properties of a Highly Ordered Hybrid Interface: The Case of Nickel Tetra-Phenyl-Porphyrins on Fe(001)–*p*(1 × 1)O

**DOI:** 10.3390/mi12020191

**Published:** 2021-02-13

**Authors:** Guglielmo Albani, Alberto Calloni, Andrea Picone, Alberto Brambilla, Michele Capra, Alessandro Lodesani, Lamberto Duò, Marco Finazzi, Franco Ciccacci, Gianlorenzo Bussetti

**Affiliations:** Physics Department, Politecnico di Milano, Piazza Leonardo da Vinci, 32, 20133 Milano, Italy; alberto.calloni@polimi.it (A.C.); andrea.picone@polimi.it (A.P.); alberto.brambilla@polimi.it (A.B.); michele.capra@polimi.it (M.C.); alessandro.lodesani@polimi.it (A.L.); lamberto.duo@polimi.it (L.D.); marco.finazzi@polimi.it (M.F.); franco.ciccacci@polimi.it (F.C.); gianlorenzo.bussetti@polimi.it (G.B.)

**Keywords:** hybrid interfaces, photoemission spectroscopy, thin films

## Abstract

In this paper we focus on the structural, electronic, and magnetic properties of Ni tetra-phenyl-porphyrins (NiTPP) grown on top of Fe(001)–*p*(1 × 1)O. Ordered thin films of metal TPP molecules are potentially interesting for organic electronic and spintronic applications, especially when they are coupled to a ferromagnetic substrate. Unfortunately, porphyrin layers deposited on top of ferromagnetic substrates do not generally show long-range order. In this work, we provide evidence of an ordered disposition of the organic film above the iron surface and we prove that the thin layer of iron oxide decouples the molecules from the substrate, thus preserving the molecular electronic features, especially the HOMO-LUMO gap, even when just a few organic layers are deposited. The effect of the exposure to molecular oxygen is also investigated and an increased robustness against oxidation with respect to the bare substrate is detected. Finally, we present our results for the magnetic analysis performed by spin resolved spectroscopy, finding a null magnetic coupling between the molecules and the substrate.

## 1. Introduction

During the last decade, much effort has been devoted to produce spintronic devices to flank standard silicon-based technology [1]. In parallel to studies concerning metals and semiconductors [2], scientific research has also focused on hybrid organic–inorganic interfaces [3,4], triggered by the possibility to scale the dimensions of a device down to the single molecule [5,6]. Moreover, ab-initio molecular dynamics has become increasingly available for addressing nucleation, patterning, and bonding in such systems [7,8]. A typical goal in organic electronics consists in preserving some of the features of the free standing molecules, such as the energetic positions of the highest occupied molecular orbital (HOMO) and the lowest unoccupied molecular orbital (LUMO) [9,10], when the molecules are deposited onto a substrate. Furthermore, in view of the potential application of these molecules in a device, an ordered, directly accessible layer of molecules, with a certain resistance against gaseous contaminants [11], would be equally important. A further essential feature that molecules grown on top of crystalline substrates must possess for spintronic applications is that they should carry a magnetic moment [12,13].

Among the vast choice of molecular units, we decided to focus our attention on metallo-tetra-phenyl-porphyrins (MTPP), which can host a transition metal (TM) ion in the inner ring. Tetra-phenyl-porphyrins are very well known compounds thanks to their relatively easy synthesis [14], their thermal stability [15], and for their applications in photovoltaic [16,17], sensing [18,19], and spintronics [12,13]. The properties of these molecules have been studied by depositing them on top of insulating [20], semiconductive [21] and conductive substrates [22]. Their open structure, with the phenyls not folded up on the macroring, allows to directly address the inner TM ion [23]. However, a severe drawback is the possibility for the molecule to strongly interact with the surroundings. Therefore, in order to preserve the molecular features, they are usually grown on top of noble metals [22] or organic crystals [24]. Another strategy consists in decoupling the molecular film from the substrate underneath with a graphene layer [25] or with a thin layer of metal oxide [26]. This solution enables the possibility to employ diverse metallic substrates, which are essential not only for the realization of electric contacts but also to expand the possible magnetic applications of the system.

Since we are interested in the study of the magnetic coupling of the molecular layer with the substrate, we employed a thick film (≈500 nm) of iron grown onto MgO(001). This guarantees a high crystal quality of the iron substrate [27,28,29]. To decouple the organic film from the metallic substrate, we passivated it with a ultra-thin layer of iron oxide, namely Fe(001)–*p*(1 × 1)O [30]. MTPPs on Fe(001)–*p*(1 × 1)O have already been reported as interesting examples of weakly interacting hybrid interfaces, due to the ultra-thin oxidation of the substrate [26]. With the perspective to fabricate a nano-scale device, it is very important to check the local structure of the system. The goal is to demonstrate the presence of a regular array of molecules on top of the Fe–*p*(1 × 1)O substrate, as already reported for ZnTPP [29]. In this system, the central TM ion has the 3*d* shell fully occupied, and the electronic features of the molecules, such as the HOMO-LUMO gap, are preserved when they are grown onto the substrate [31]. Moreover, an ordered structure of the single molecule film is obtained [32]. In this paper, we present our results for the system NiTPP/Fe–*p*(1 × 1)O, in order to understand whether (i) the ordering of the molecular layer is still preserved, (ii) the electronic features are maintained, (iii) the system is robust against gaseous contaminants, and (iv) the choice of Ni as the inner ion is effective to introduce a magnetic response of the system introducing an orientation of the magnetic moments carried by the molecules, with a detailed analysis expanding our preliminary results [33].

## 2. Materials and Methods

The preparation and the analysis of the sample were performed in situ, in ultra-high vacuum (UHV) conditions with pressure in the high 10^−11^ Torr regime [34]. The surface of a thick Fe film (thickness ≈ 500 nm) grown on a MgO(100) substrate was cleaned by a series of cycles of Ar^+^ sputtering followed by low-temperature (480 °C) annealing [11]. To obtain a clean Fe–*p*(1 × 1)O termination, the sample was exposed to 30 L of oxygen (with 1 L = 10^−6^ Torr·s) and then to a higher temperature annealing (700 °C) to remove the excess of oxygen. The quality of the surface was then verified with low energy electron diffraction (LEED), scanning tunneling microscopy (STM) and ultraviolet photoemission spectroscopy (UPS) [30,35,36]. The molecules (from Merck KGaA, Darmstadt, Germany) were purified in vacuum and deposited in a dedicated chamber from a quartz crucible. The deposition rate was measured with a quartz microbalance and maintained around 1 Å/min [37]. In the following, the deposited thickness is expressed in monolayers (ML), with 1 ML = 3.06 Å. The sample was maintained at room temperature (RT) during deposition.

STM measurements were acquired in UHV conditions using a commercial Omicron Variable Temperature system (Scienta Omicron, Uppsala, Sweden). STM images were obtained at room temperature (RT) in constant-current mode with W tips electrochemically etched in a NaOH solution. X-ray photoemission spectroscopy (XPS) and UPS were performed with a Mg anode X-ray lamp (*E*_ph_ = 1253.6 eV) and an UV lamp exploiting the HeI emission line (*E*_ph_ = 21.2 eV), respectively. Photoemitted electrons were analyzed with a 150 mm hemispherical electron analyzer from SPECS GmbH (SPEC^TM^, Berlin, Germany). Spin resolved measurements (SP-UPS) were performed with the sample magnetized in remanence, with a coil providing a pulsed field of 1000 Oersted. Spin polarized electrons were detected by a Mott polarimeter with thorium target. Inverse photoemission spectroscopy (IPES) measurements were performed with electrons excited by a GaAs diode laser (λ = 808 nm) illuminating a GaAs target treated to exhibit a negative electron affinity condition. IPES spectra were measured in the isochromatic mode by varying the energy of the electrons impinging on the sample and selecting the outgoing photons with a band pass filter centered at 9.6 eV. The total full width at half-maximum (FWHM) resolution of the apparatus is 0.9 eV for XPS, 0.05 eV for UPS, 0.15 eV for SP-UPS [11], 0.6 eV for IPES [38,39].

## 3. Results and Discussion

### 3.1. Structure and Morphology

**Figure 1:**
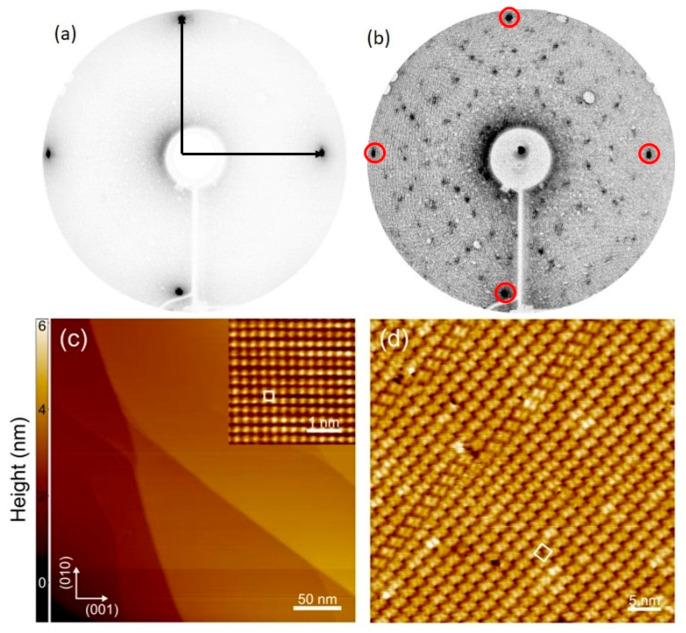
(**a**) LEED image acquired image on the Fe–*p*(1 × 1)O substrate. Black arrows indicate a basis of the reciprocal lattice. (**b**) LEED image acquired on the 1 ML NiTPP sample. The beam energy in both panels is 55 eV. Red circles indicate the spots due to the substrate. (**c**) Large scale STM image of the Fe–*p*(1 × 1)O substrate. Tunneling parameters are *V* = 1 V, *I* = 1 nA, image size is 300 × 300 nm^2^. The inset shows an atomically resolved image of the surface, acquired with *V* = 0.1 V, *I* = 10 nA tunneling parameters. The white square indicates the surface unit cell, with a lattice parameter of 0.29 nm. (**d**) The 32.6 × 32.6 nm^2^ STM image acquired on the 1 ML NiTPP sample. The white square indicates the unit cell of the (5 × 5) R 37 reconstruction. Tunneling parameters are *V* = 2 V, *I* = 500 pA.

The results acquired by Low Energy Electron Diffraction (LEED) and STM are shown in Figure 1. 

Figure 1a displays the LEED pattern of the bare Fe–*p*(1 × 1)O substrate. The periodicity of the Fe(001) surface is not modified by passivation, as reported in literature [35,40,41]. When a single layer of NiTPP is deposited on top of the surface, the periodicity of the system is modified, but a regular pattern is still visible, as shown in Figure 1b. The molecular arrangement is compatible with a (5 × 5) commensurate pattern, as already reported for ZnTPP [32]. The LEED pattern suggests the presence of two domains, rotated by 37° (R 37), similarly to the pattern assumed by CoTPP on Fe–*p*(1 × 1)O [11,26]. This symmetry is mostly evident at 1 ML coverage. As the thickness grows, the LEED pattern starts to fade and at 2 ML coverage it has completely disappeared. 

Figure 1c displays a constant current STM image acquired on the Fe–*p*(1 × 1)O substrate before NiTPP deposition. At mesoscopic scale, the surface is characterized by atomically flat terraces separated by multiatomic steps. As discussed in the literature [41], the oxygen overlayer promotes the step bunching and increases the terrace width with respect to the oxygen-free Fe(001) surface, providing an ideal template for the molecular self-assembly. The atomically resolved image shown in the inset of Figure 1c reveals the square lattice of the Fe–*p*(1 × 1)O surface, with a lattice constant of about 0.29 nm. Figure 1d displays the surface topography after deposition of 1 ML of NiTPP molecules. In agreement with LEED measurements, the molecular overlayer forms a (5 × 5) R 37 reconstruction with respect to the substrate. Besides the information about the translational symmetry, STM measurements provide further structural details about the molecular film. In the first place, the molecules form a compact overlayer with domains extending over tens of nanometers. Notice that in Figure 1d only one of the two equivalent rotational domains is visible. Secondly, the single molecule can assume different azimuthal orientation inside the square unit cell, nevertheless preserving a long-range order.

The ordered commensurate arrangement of the molecular array is probably associated with the flatness of the substrate and with the reduced interaction of the molecules, with the latter as a consequence of the presence of the thin oxide layer. More specifically, a reduced interaction should correspond to a higher mobility of the molecules on top of the substrate, fostering their arrangement in ordered domains.

### 3.2. Electronic Structure

**Figure 2:**
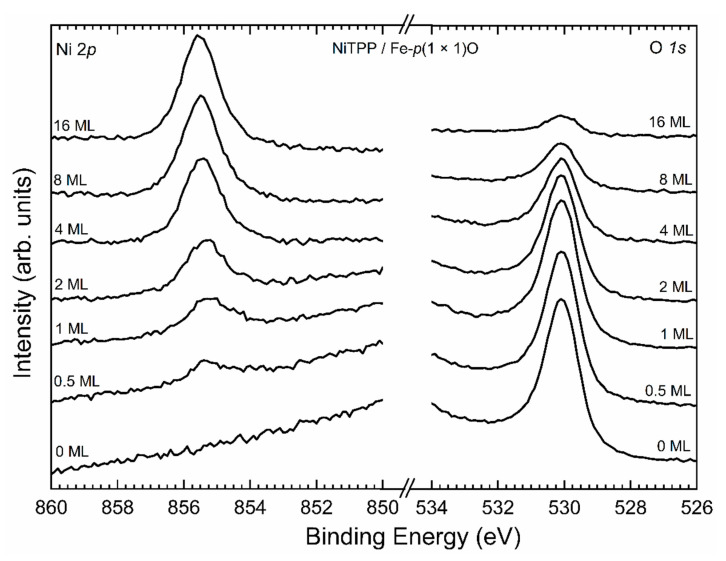
XPS spectra acquired with Mg Kα emission line (*hν* = 1253.6 eV) in the Ni 2*p* (left) and O 1*s* (right) regions for the system NiTPP/Fe–*p*(1 × 1)O. The spectra are displayed from bottom to top with growing thickness of the NiTPP layer.

Since it has been demonstrated that oxygen can float when thin films are deposited on top of Fe–*p*(1 × 1)O [42], we performed an XPS experiment to understand whether oxygen remains buried under the organic layer maintaining its decoupling effect or not. XPS spectra are shown in Figure 2.

As the thickness of the organic layer increases, the signal from the Ni ions belonging to the molecules becomes more evident, while oxygen starts to decrease and it remains just as a small trace when a 16 ML coverage is reached. The expected O 1*s* signal attenuation is about 90% when a film of 16 ML (5 nm) is studied, according to the literature [43]. Therefore, oxygen atoms remain buried between the molecules and the substrates and they cannot float on top of the system, as they do when metallic layers are deposited on top of the same substrate [42]. This is probably due to the fact that it is not energetically convenient for the oxygen atoms to break the Fe-O bonds and attach themselves to the molecules.

**Figure 3:**
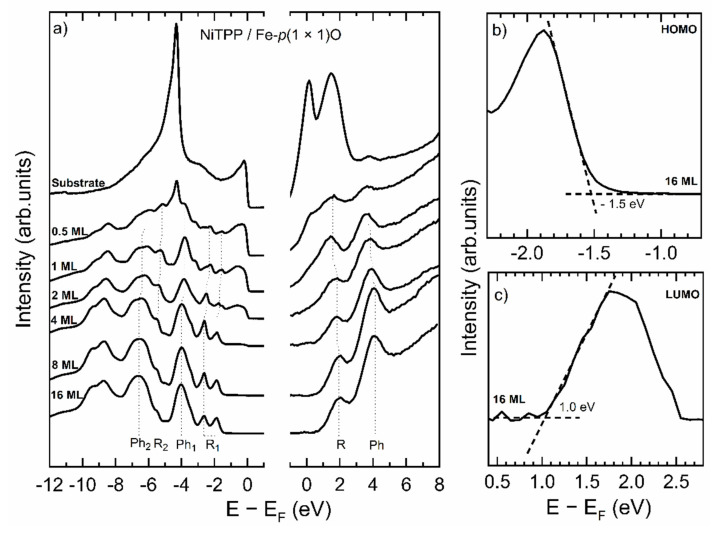
(**a**) UPS (left) and IPES (right) spectra acquired on the system NiTPP/Fe–*p*(1 × 1)O. The spectra are positioned from top to bottom at increasing NiTPP thickness. Dot lines highlight the evolution of the energy position of peaks attributable either to the macroring (R, R_1_ and R_2_) or to the phenyl groups (Ph, Ph_1_ and Ph_2_). (**b**) Energy region around the HOMO for the 16 ML NiTPP/Fe–*p*(1 × 1)O sample. The HOMO energy is evaluated as the interception between the zero line and the tangent line to the rising edge in the inflection point (dash lines). (**c**) Energy region around the LUMO for the 16 ML NiTPP/Fe–*p*(1 × 1)O sample. A linear background was subtracted to the peak. The LUMO energy is evaluated as the interception between the zero line and the tangent line to the rising edge in the inflection point (dash lines).

According to the literature [31], when the thickness of the organic layer exceeds 4 ML any electronic interaction with the substrate becomes essentially negligible. Thus, a comparison between the spectra of thin (0.5 ML, 1 ML) and thick (8 ML, 16 ML) films will provide information about the consequences of interactions between the substrate and the molecules. In Figure 3, the evolution of filled and empty states around the Fermi edge is reported.

Figure 3a displays the spectra acquired by UPS and IPES on the system as a function of the thickness on the molecular layer. The substrate displays a dominant peak at a binding energy (E − E_F_) around −5 eV, which is related to oxygen. This peak is still visible at 0.5 ML coverage, but is completely quenched when a uniform coverage with 1 ML thickness is completed. At this point, all the spectral features of the molecules are well visible. In Figure 3a they are labeled according to the corresponding region in the molecule corresponding to each specific peak. The majority of the features are due to the central pyrrolic macroring (labeled with “R” in Figure 3a) and to the four peripheral phenyls (labeled with “Ph”). It is quite evident that the only modifications that occur in the spectra, going from 1 to 16 ML are small shifts in the positions of the peaks or slight variations of the relative intensity, thus conveying the message that the molecules are decoupled from the substrate even at 1 ML coverage. 

To strengthen this conclusion, we evaluated the HOMO-LUMO gap of the samples. The HOMO and the LUMO energies are determined by evaluating the rising edges of the respective features, as shown in Figure 3b,c [23,44]. To help the correct evaluation of the LUMO, a linear background was subtracted. The results of the evaluation of the HOMO-LUMO gap are displayed in Table 1, with an uncertainty of ±0.2 eV for the less covered samples and ± 0.1 eV for the other ones. This analysis confirms that not only the HOMO-LUMO gap is still present when thin layers of NiTPP are deposited on Fe–*p*(1 × 1)O, but also that its value is preserved. The small variation is due to the more efficient screening of the photogenerated hole on the single layer film [23]. The energy position of the HOMO and the LUMO plays an important role in view of possible applications. In hybrid electronic devices it determines the band alignment between the organic system and the metal electrodes, while in optical devices the HOMO-LUMO gap sets the transparency window. From the data reported in the table, a clear change in the energy gap occurs above the 4th monolayer (in good agreement with previous data [32]), which suggests that the substrate is completely screened after this coverage.

### 3.3. Stability against Gaseous Contaminants

**Figure 4:**
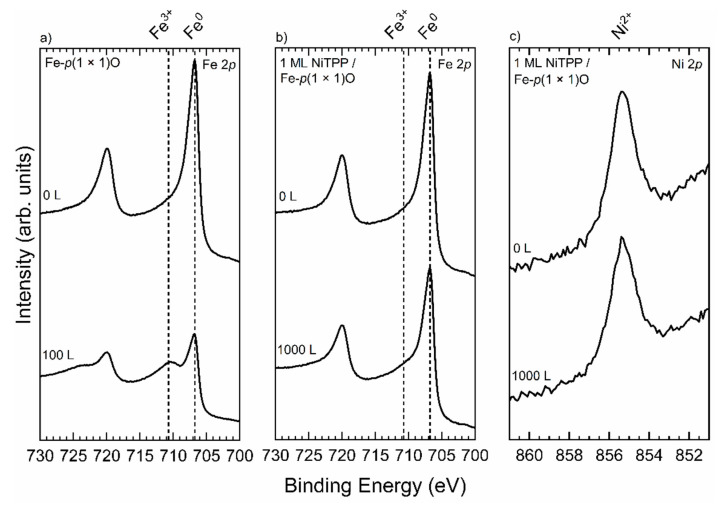
(**a**) Fe 2*p* XPS spectra of the Fe–*p*(1 × 1)O surface before and after exposure to 100 L of O_2_. (**b**) Fe 2*p* XPS spectra of 1 ML NiTPP/Fe–*p*(1 × 1)O before and after exposure to 1000 L of O_2_. In both the panels (**a**) and (**b**) the two vertical dashed lines mark the energy position of Fe^0^ and Fe^3+^-derived peaks. (**c**) Ni 2*p* XPS spectra of 1 ML NiTPP/Fe–*p*(1 × 1)O before and after exposure to 1000 L of O_2_. All the samples were exposed to molecular oxygen at RT. All the spectra were acquired with Mg Kα (*hν* = 1253.6 eV) emission line.

We exposed the 1 ML-thick sample and the Fe(100) surface to molecular oxygen. The results of the comparison are shown in Figure 4.

Figure 4a displays the evolution of the XPS spectrum of Fe–*p*(1 × 1)O in the Fe 2p region. When the sample is exposed to 100 L of O_2_ a shoulder peak arises in a position compatible with that typical of Fe^3+^-derived peaks [45,46]. This indicates that an exposure of 100 L is enough to massively oxidize the surface. When 1 ML of NiTPP is deposited onto the fresh Fe–*p*(1 × 1)O substrate and the whole system is again exposed to oxygen, the situation changes dramatically. Indeed, as it is shown in Figure 4b, no features related to the presence of Fe^3+^ appear even at 1000 L exposure. Furthermore, Figure 4c indicates no variations in the position of the Ni 2*p* peak and this denotes that the oxidation state of this ion is not modified. These results convey the information that molecular oxygen, at this exposure, does not stick to the molecules and cannot penetrate the molecular layer to further oxidize the substrate underneath, thus suggesting a protecting effect of the organic layer [11].

### 3.4. Magnetic Analysis

**Figure 5:**
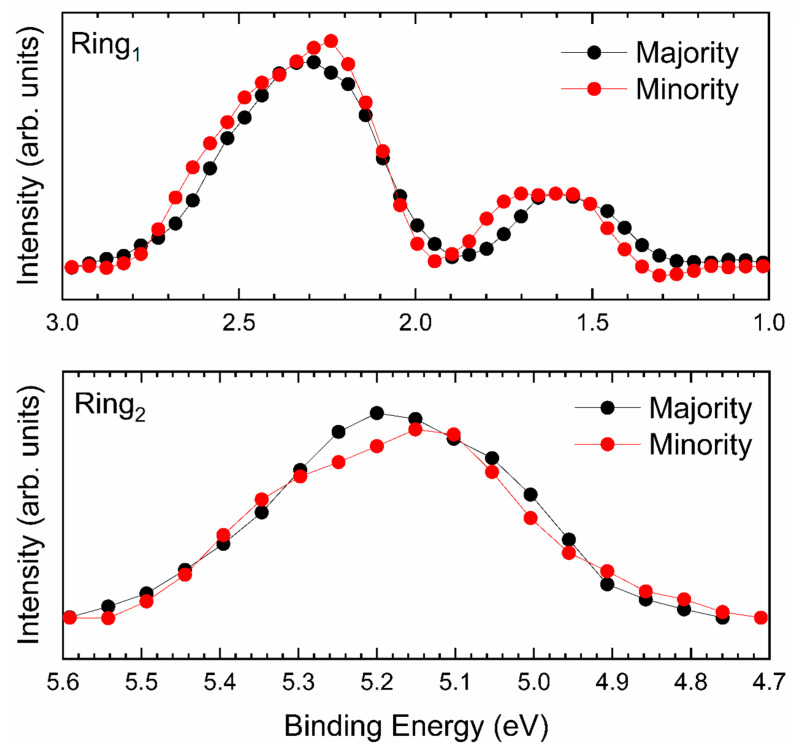
SP-UPS spectra of the system 1 ML NiTPP/Fe–*p*(1 × 1)O in the energetic regions “Ring_1_” (upper panel) and “Ring_2_” (lower panel). In black (red) the spectra related to the majority (minority) spin populations are reported. A linear background was subtracted in both panels.

After having magnetized the substrate [47], we focused our spin-resolved analysis in the energetic regions with features assigned to the ring, as highlighted in Figure 3. The results of the magnetic analysis of the sample are shown in Figure 5.

Both the upper and the lower panel of Figure 5 do not show any unbalance between the majority and minority spin populations, thus suggesting the impossibility for the molecule to magnetically couple to the substrate. Thus, we have to conclude that, within our experimental resolution, we cannot give any evidence of an ordered magnetic moment associated to Ni^2+^ ion [33].

## 4. Conclusions

We focused on the structural, electronic and magnetic properties of the system NiTPP/Fe–*p*(1 × 1)O. We showed that a single layer of NiTPP is arranged in an ordered commensurate (5 × 5) superpattern above the substrate. This organic array is organized in large domains with two possible orientations rotated by 37°. This ordering is possible thanks to the decoupling effect of the thin layer of iron oxide. Consequently, this system can be regarded as an interesting template for the construction of regular molecular structures [48]. The thin oxide layer is also responsible for the preservation of the electronic structure of the NiTPP system. Indeed, the electronic properties of the latter are preserved even at a single layer coverage, thus making the substrate passivation by an ultra-thin oxide layer very promising in view of applications in the field of organic electronics. Moreover, we demonstrated that a single layer of NiTPP protects the Fe–*p*(1 × 1)O surface at least up to 1000 L of O_2_ exposure, thus making the system more robust against the effect of gaseous contaminants. Finally, we focused on the magnetic properties of the system, looking for any evidence of a magnetic coupling between 1 ML of NiTPP and the substrate. The results go in the direction of the absence of a magnetic moment carried out by the molecules.

## Figures and Tables

**Table 1:** HOMO-LUMO calculated for the system NiTPP/Fe–*p*(1 × 1)O as a function of the thickness of the deposited molecular film.

NiTPP Coverage	Energy Gap
0.5 ML	2.0 ± 0.2 eV
1 ML	2.0 ± 0.2 eV
2 ML	2.1 ± 0.1 eV
4 ML	2.3 ± 0.1 eV
8 ML	2.5 ± 0.1 eV
16 ML	2.5 ± 0.1 eV
